# HIV-1 Accessory Proteins Adapt Cellular Adaptors to Facilitate Immune Evasion

**DOI:** 10.1371/journal.ppat.1003851

**Published:** 2014-01-23

**Authors:** David R. Collins, Kathleen L. Collins

**Affiliations:** Department of Microbiology and Immunology, University of Michigan, Ann Arbor, Michigan, United States of America; Columbia University, United States of America

## Viral Strategies to Overcome Host Barriers to Infection

As obligate intracellular parasites, viruses must gain entry into target cells and utilize the host cellular machinery for production of viral progeny. After entering the cell and localizing to an intracellular niche, the virus sheds its capsid, replicates its genome, transcribes its RNA, translates its protein components, and assembles the components to form new progeny virions that can infect new cells. At each stage of the virus life cycle, the host has evolved mechanisms to restrict successful infection, and pathogenic viruses have evolved countermechanisms to overcome each restriction. This article will focus on the mechanisms by which the lentivirus human immunodeficiency virus type 1 (HIV-1) overcomes these barriers to establish and propagate infection in human cells.

## Lentiviruses Encode Accessory Proteins that Counteract Host Antiviral Responses

While all retroviruses encode proteins required for entry, reverse transcription, integration into host DNA, protein processing, capsid formation, and genome packaging (Env, Pol, and Gag); more complex retroviruses of the lentivirus family, including HIV-1, encode several additional genes. Two of these genes encode proteins that regulate transcription and mRNA nuclear export (Tat and Rev respectively). The remaining genes (*nef*, *vif*, *vpu*, *vpr*, and/or *vpx*) encode “accessory proteins” that are not always required for viral infection in in vitro cell culture systems. Instead, these proteins enable pathogenesis in vivo by allowing lentiviruses to evade antiviral responses. Accessory proteins presumably evolved their functions under the selective pressures of continual replication in primate hosts, with each factor serving at least one specific role to enhance viral fitness. Decades of HIV-1 research have led to several key breakthroughs in our understanding of the specific activities and functions of accessory proteins. Interestingly, each accessory protein functions as an adaptor between two or more known host cellular proteins. In this way, the viral pathogen succeeds in dramatically enhancing its capacity to alter the host environment while minimizing its genome size.

## HIV-1 Nef Adapts Clathrin Adaptors to Evade Cytotoxic T Lymphocytes (CTLs) and Promote Viral Spread

**Figure 1:**
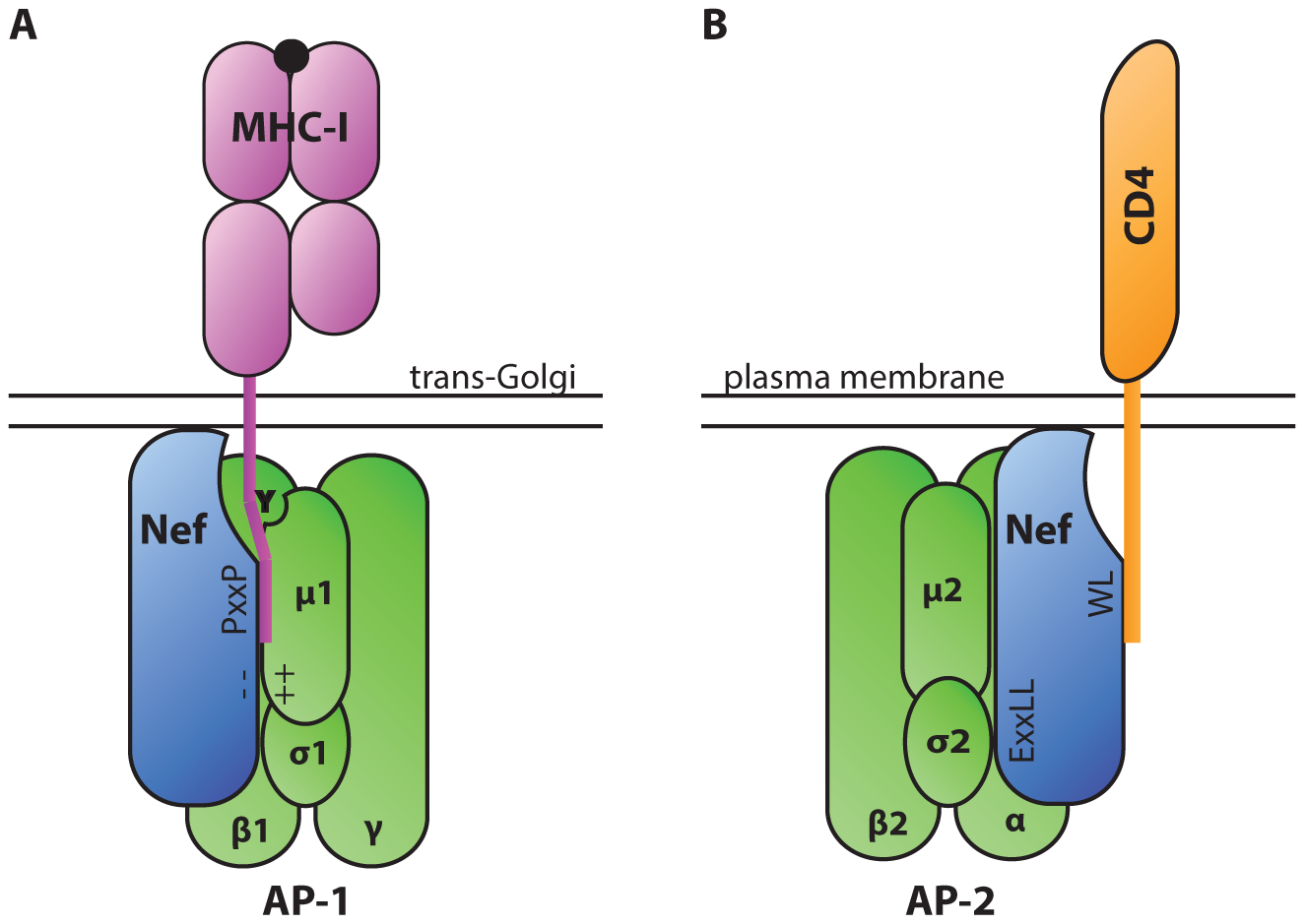
Diagrammatic representation of HIV-1 Nef serving as an adaptor of clathrin adaptor proteins in cellular trafficking pathways. A. The AP-1 µ1 subunit interacts with a tyrosine residue in the cytoplasmic tail of MHC-I via a tyrosine-binding pocket. This interaction is stabilized by electrostatic interactions between a poly-glutamic acid motif of Nef (− −) and a positively charged patch in AP-1 µ1 (++). A polyproline repeat of Nef (PxxP) further stabilizes the complex by forming a wall of the groove that contains the MHC-I tail. These interactions lead to down-modulation of MHC-I from the cell surface. B. AP complexes interact with Nef via a dileucine motif (ExxLL) in the Nef C-terminal loop. AP complexes bind dileucine motifs at an interface between the AP complex σ and heavy chain subunits (α, β, or γ in AP-2, AP-3, and AP-1 respectively) [30]. Nef utilizes the dileucine trafficking signal to down-modulate a number of host proteins, including CD4.

To establish a successful infection, intracellular pathogens must evade CTLs, which recognize foreign antigens presented in association with host major histocompatibility complex class I (MHC-I). One way that HIV-1 achieves this goal is through the activity of the accessory protein negative effector factor (Nef). Nef enhances the survival of infected cells in the presence of CTLs by mislocalizing and degrading MHC-I [1], [2]. To accomplish this, Nef stabilizes an interaction between MHC-I and the clathrin adaptor protein-1 (AP-1), which regulates clathrin-dependent trafficking of proteins between the trans-Golgi network and endosomes. When stabilized in this complex by Nef, AP-1 directs MHC-I to the endolysosomal pathway where it is degraded at an accelerated rate [3]. Biochemical and structural analysis have revealed that a critical tyrosine residue in the MHC-I cytoplasmic tail mediates the interaction with the tyrosine-binding pocket in the µ1 subunit of AP-1 [4]–[6] (Figure 1A). While this tyrosine can weakly bind AP-1 in some cell types [7], a complex containing MHC-I and AP-1 is normally not detected in T lymphocytes. This is primarily because the MHC-I cytoplasmic tail tyrosine does not conform to a canonical AP-1 tyrosine signal in which there is a downstream hydrophobic amino acid (Yxxφ). Nef stabilizes the weak interaction between MHC-I and AP-1 by providing additional contacts with AP-1 and with the MHC-I cytoplasmic tail. Specifically, an acidic cluster in Nef forms an electrostatic interaction with positively charged residues of AP-1 µ1 [6]. In addition, polyproline (PxxP) repeats in Nef lock the MHC-I cytoplasmic tail onto µ1 (Figure 1) [6].

Interestingly, Nef also interacts directly with clathrin adaptor proteins AP-1, AP-2, and AP-3 through a canonical dileucine trafficking signal in Nef's C-terminal loop domain (reviewed in [8]). By simultaneously binding to host protein cytoplasmic tails and clathrin adaptor proteins, Nef facilitates the down-modulation of a number of host proteins from the cell surface, including CD4 (Figure 1B) [9]. Down-modulation of CD4, the main cellular receptor for HIV entry, enables HIV-1 to avoid Env-CD4–mediated retention of virions at the cell surface and promotes efficient virus release and dissemination [10], [11].

## HIV-1 Vif, Vpu, and Vpr Adapt Cellular Ubiquitin Ligase Adaptors to Counteract Host Antiviral Responses

**Figure 2:**
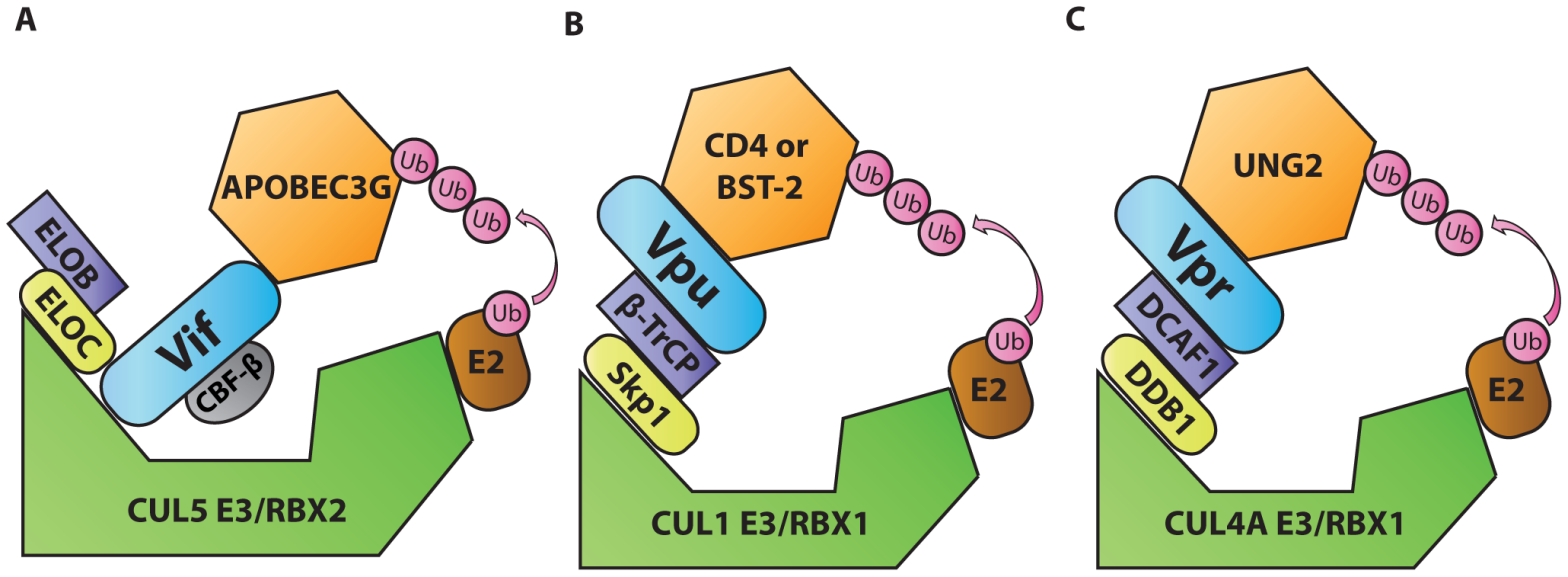
Diagrammatic representations of HIV-1 Vif, Vpu, and Vpr functioning as adaptors of substrate adaptors in cellular ubiquitination pathways. A. Vif, in complex with and stabilized by cellular CBF-β, binds to the EloBC/Rbx2/Cullin5 E3 ubiquitin ligase complex and to APOBEC3G to induce its polyubiquitination and degradation. B. Vpu interacts with the Skp1/Cullin/F-Box (SCF) ubiquitin ligase complex via β-TrCP and with target proteins BST-2 or CD4 to induce their ubiquitination and mislocalization. C. Vpr interacts with the DCAF1/DDB1/Rbx1/Cullin4A E3 ubiquitin ligase complex and with UNG2 to induce its polyubiquitination and degradation.

Ubiquitination is a post-translational protein modification that regulates protein degradation and trafficking. Cellular E3 ubiquitin ligases facilitate the transfer of ubiquitin from E2 ubiquitin-conjugating enzymes to lysine, serine, or threonine residues on specific target proteins. E3 ligases often comprise multi-protein complexes that include a scaffold, an adaptor, and a target protein substrate. By serving as substrate adaptors that simultaneously interact with ubiquitin ligase adaptors and cellular target proteins, three HIV accessory proteins (Vif, Vpu, and Vpr) induce ubiquitination of host targets. This leads to proteasomal degradation and/or mislocalization of targeted host proteins. For example, viral infectivity factor (Vif), an accessory protein encoded by primate lentiviruses, including HIV-1, counteracts the antiviral activities of apolipoprotein B mRNA editing complex 3 (APOBEC3, or A3) proteins, especially APOBEC3G (A3G) [12]. A3 deaminases, which attack single-stranded DNA converting cytidine to uridine, have broad antiviral functions (reviewed in [13]). In the absence of Vif, A3G-mediated cytidine deamination results in uridination of the first strand of DNA synthesized by the viral reverse transcriptase. Guanosine-to-adenosine hypermutation results as uridine residues are paired with adenosine upon second strand synthesis. There is also evidence that A3G has a separate inhibitory effect on the processivity of reverse transcription (reviewed in [13]). In HIV-1-infected T cells, A3G activity can induce a DNA damage response that stimulates up-regulation of natural killer (NK) cell-activating ligands on the surface of the infected cells and activates NK cell lysis of infected cells [14]. To evade A3-mediated responses, the HIV-1 Vif protein simultaneously binds A3G and the ubiquitin ligase adaptor EloBC, causing polyubiquitination by the Rbx2/Cullin5 E3 ubiquitin ligase complex (Figure 2A) [15]. An additional cellular protein, core binding factor β (CBF-β), stabilizes the formation of this complex [16], [17]. By driving the ubiquitin-dependent degradation of A3 family members, Vif enables viral escape from A3-mediated antiviral restriction. The critical importance of A3G as a cellular factor that restricts lentiviruses is evidenced by coevolution of Vif and A3G sequences [18].

Another example of this tactic is displayed by HIV-1 viral protein U (Vpu). This accessory protein promotes virus release by counteracting the antiviral activities of the interferon-induced restriction factor bone marrow stromal antigen 2 (BST-2/tetherin/CD317/HM1.24) [19], [20]. Vpu also down-modulates the HIV-1 receptor CD4 (reviewed in [21]). In the absence of Vpu, CD4 and BST-2 inhibit the release of infectious viral particles. CD4 binds virions through interactions with Env glycoproteins, and BST-2 tethers virions by virtue of its unusual structure. The general consensus of a number of studies is that BST-2 is attached to membranes via its transmembrane domain at its C-terminus and via its N-terminal glycophosphatidylinositol anchor. By simultaneously binding to viral and cellular membranes, BST-2 tethers virions, preventing their release. To evade BST-2, Vpu acts as an adaptor that promotes an interaction between a ubiquitin ligase substrate adaptor [beta transducing repeat-containing protein (β-TrCP)] and target proteins. In this way, Vpu promotes ubiquitination of the target protein by Skp1/Cullin1/F-box (SCF) ubiquitin ligase complex (Figure 2B) ([22] and reviewed in [21]). Recent studies have expanded the role of BST-2 to include viral sensing and signal transduction to activate NF-κB–dependent pro-inflammatory signals (reviewed in [23]). Like A3, the significance of BST-2–mediated restriction is illustrated by co-evolution of lentiviral genomes with species-specific variations in BST-2. In this regard lentiviruses have demonstrated remarkable flexibility. While HIV-1 utilizes Vpu to target BST-2, most primate lentiviruses use Nef for this purpose, and still others can use Env (reviewed in [21]). In this way, BST-2 variation appears to serve as a barrier to cross-species infection.

Viral protein R (Vpr) is a pleiotropic lentiviral accessory protein that has been shown to activate the DNA damage response, up-regulate NK activating ligands, cause cell-cycle arrest, and promote infection of macrophages (reviewed in [24]). Like Vif and Vpu, Vpr adapts a substrate adaptor of a cellular ubiquitin ligase complex [damaged DNA binding protein 1-cullin 4-associated factor 1 (DCAF1)], promoting ubiquitination by a ubiquitin ligase complex (Rbx1/Cullin4A E3, Figure 2C, reviewed in [24]). One protein targeted by Vpr is the cellular uracil DNA glycosylase 2 (UNG2) [25]. However, the precise role of UNG2 remains controversial as it has both positive and negative effects on HIV-1 replication (reviewed in [26]). Because the interaction with UNG2 does not appear to explain all of Vpr's activities, it is likely that Vpr targets additional cellular proteins that have not yet been identified. A structurally related accessory protein, viral protein X (Vpx), which is encoded by HIV-2 and some viruses of the simian immunodeficiency virus (SIV) family, also interacts with the DCAF1 to promote the degradation of a cellular nucleotide triphosphate phosphohydrolase [SAM domain and HD domain-containing protein 1 (SAMHD1)] [27]. In the absence of Vpx, SAMHD1 inhibits reverse transcription by depleting the intracellular pool of deoxynucleoside triphosphates [28]. Vpx-mediated polyubiquitination of SAMHD1 induces its proteasomal degradation and allows viral replication in myeloid cells [27]. Like the other pairs of viral accessory protein and cellular targets described thus far, Vpx has co-evolved with SAMHD1 from different primate species, acquiring the capacity to utilize different SAMHD1 molecular interfaces to promote its degradation [29].

## Accessory Proteins as Tools for Identifying Important Antiviral Defense Mechanisms

The continual battle between host and virus provides constant selective pressure that shapes the viral genome. Research focused on the specific interactions that have evolved between lentiviral accessory proteins and their cellular targets has led to the identification and characterization of several antiviral factors (A3G, BST-2, and SAMHD1) and has informed our understanding of MHC-I trafficking pathways. Importantly, these host factors have broad and significant antiviral effects that can restrict a diverse array of viruses in addition to lentiviruses. The study of interactions between viral and host proteins is likely to continue to yield new information about important host defenses that may facilitate the development of improved treatments for a variety of human diseases.
